# A Street Ambulance Service for London

**Published:** 1889-04-06

**Authors:** 


### A Street Ambulance Service for London.

A goodly number of more or less philanthropic persons assembled at the Middlesex Hospital the other evening, under the presidency of Sir John Heron Maxwell, to hear a paper by Mr. Thomas Ryan, secretary to St. Mary's Hospital, on the question of "Ambulancing" the metropolis. The paper read was in Mr. Ryan's very characteristic style; crisp, trenchant, expository, illustrative, and thoroughly well drawn from the facts. There was a case to be proved, and Mr. Ryan proved it. He showed that in no part of London was there anything like adequate ambulance accommodation. He proved, moreover, that the want of such accommodation gave rise to an indescribable amount of perfectly avoidable suffering, and to dangers of the gravest kind. It was seen that sometimes deaths occurred in young and hitherto healthy persons for no other reason than that caused by the painful method of their removal to the hospital. It was not enough, however, to prove the existence of evils and dangers. There was an urgent necessity for some practical scheme whereby such evils and dangers might be dealt with and minimised. The subject could not have been entrusted to better hands than those of Mr. Ryan. He had gathered his facts so well, and arranged his order of procedure so simply and thoroughly, that everybody at once said "Amen" to his proposals. No district must be left without its ambulance, argued the speaker. Every district must be ambulanced thoroughly and adequately according to the numbers of its population and its liability to accidents. Of course, the complete carrying out of a scheme of ambulance would be expensive if stations were to be created, and men employed specially to take charge of each station. But the stations are already here. The different police stations are supplied with litters at the present time; but fire stations and railway stations abound in London, and the officials at those places are perfectly willing to enter into a scheme which may be of life-saving service to themselves as well as to other people. In fact, there seems to be no difficulty in obtaining suitable ambulance stations in London free of cost. By means of a map, which he had prepared himself, Mr. Ryan showed where ambulances are at present kept and where they are deficient. Utilising his map, he proposed to mark out points where ambulances are still required, and to supply them at once with the convenient, comforting, and inexpensive two-wheel Ashford litter. For the modest sum of £1,500 he had calculated that London could be supplied with all it needed at the present moment.

There could not have been a more sympathetic or unanimous audience. Many who had gone to the meeting fearing that a scheme would be proposed which would overwhelm them with its magnitude and its costliness were both relieved and delighted to find that, whilst Mr. Ryan's plan was to be all that was desirable in the way of efficiency, the necessary outlay would be a mere bagatelle.

The Hospitals Association has shown itself a highly practical body, and has manifested a clear understanding of the nature of the functions which such a society may be expected to perform. It does not confine itself to merely debating hospital questions, however large and important such questions may be in themselves; but seeks out deficiencies in detail and administration, and sets itself to amend what is amiss, and to supply what is lacking. During its five years of existence it has earned for itself an enviable reputation as an honest and capable body. With larger resources much more might be accomplished. In the meantime, the public must see that the small means it asks for the new and great work it has taken in hand are promptly and abundantly supplied.

The discussion which followed the reading of Mr. Ryan's paper was well sustained.

Dr. B. Howard, of New York, remarked that he had devoted a number of years to the question of ambulances, and had felt intense interest in the paper. He considered, however, that the story had not been half told, and he regretted that the public did not appreciate the extent of the mortality which resulted from cases of accident being badly transported as compared with the mortality caused directly by accidents. Some years before he had read a paper on this subject before the Medical Society of London, and a London Ambulance Service was formed. The metropolis was carefully mapped out, the ambulance centres were printed, and horse ambulances were supplied. The system, however, was not nearly so complete as it was in New York, where expense was very lightly considered as compared with the saving of human life, and he certainly considered that in this paper far too much importance had been paid to the question of expense. The first question to be considered was the best means of transport, absolutely regardless of expense. Two points which were necessary were quickness of removal and medical help with the ambulances, and he suggested a closed horse ambulance with a doctor inside. He suggested that the London County Council might be induced to deal with the entire question.

Surgeon-Major Ince also referred to the admirable and perfect system of ambulances in New York, but questioned whether the means at present available were not sufficient to meet the whole of the cases of accidents which occurred. It was, however, certainly the duty of the association to bring the matter to the notice of the London County Council, whose duty it was to concentrate the offices of the police and the fire brigades, the railway stations, and the post offices of the metropolis. All these public offices should be receptacles for some mode of conveyance of the injured to hospitals. He considered there was an unnecessary refinement in the means of conveyance, and that a litter or an Indian dooley was the most proper style of ambulance.

Mr. Burdett remarked that the Hospitals Association would not be worthy of existence if it were not a practical body. It was because it was so that Mr. Ryan's paper had been read before its members that evening, and he considered he was entitled to call it a practical association when he looked back on the work it had accomplished in recent years. It was the Hospitals Association which originated the Nurses' Pension Fund, an undertaking which, owing mainly to the courage and intelligence of the women engaged in nursing in this country, promised to be one of the most remarkable and one of the greatest financial successes of the century. Then the Hospitals Association had initiated another movement—that of nurse registration—and in many other instances it had brought about excellent results. He had had an opportunity that day of considering the question of the financial cost of the proposals in Mr. Ryan's paper with gentleman who would probably find the money for the first outlay, and the question they asked was whether the County Council, which was going to put up our rates enormously, would do this work? His answer was that it might ultimately do so, but that while we were waiting for the County Council to find out what powers it possessed, and how it could most usefully exert those powers, people were unnecessarily suffering and unnecessarily dying in London. With these facts before him, he stated with great pleasure that if the £300 a year maintenance was forthcoming, certain gentlemen were prepared to give the £1,500 to lay down the plant and to pay for the first year's expenses. Human life was of the utmost importance, but human life was imperilled and often sacrificed for the want of an adequate system of ambulances in London. He expressed pleasure at the presence of Dr. Howard, an earnest worker in ambulance work, and said the question of an extension of the ambulance system of London should be dealt with at once. He hoped Mr. Ryan's proposals would be put in practice within three months' time, and he earnestly trusted that the meeting would not separate simply relying on some future effort of the County Council, but would refer the matter to the council of the Hospitals Association.

Dr. Steele said there could be no two opinions as to the advisability of improving the present very rough and clumsy means of conveying cases of accidents to the hospitals. The duty of sending out for cases of accidents should not be allowed to devolve upon the hospitals, which had already quite enough to do to accommodate the accidents which were brought to them. Ambulances ought to be stationed at all the fire stations and police stations as well as at all the refuges. The suggestion that the municipal authorities should take charge of this matter was a very good one, and, as Mr. Burdett had rightly shown, it was the duty of the Hospitals Association to take the matter up in the meantime and to refer it to a committee.

Mr. Michelli agreed as to the difficulty which hospital authorities had in reference to the removal of hospital cases, and hoped that more of the St. John's Association litters would be provided. He considered that it only remained for an effort to be made to raise this money for the scheme to be successfully carried out.

The Chairman expressed great satisfaction at the interesting paper of Mr. Ryan, and was confident that the sum required would be raised. The following resolution were carried unanimously: "That it is the opinion of this meeting that the scheme expounded in Mr. Ryan's paper is practicable and desirable. That the scheme be recommended to the council of the Hospitals Association for adoption, with a view to the establishment of an ambulance service in connection with the suggestion that a sectional committee be appointed to establish and administer the same." One gentleman, on reading the leader in *The Hospital* last week, at once subscribed £100. The Council of the Hospitals Association appointed a committee to work the scheme on Thursday week, and on Tuesday the whole of the £1,500 was paid in. The ambulance committee consists of Sir S. Waterlow, Bart., and Messrs. Bischoffsheim, Burdett, Michelli, Ryan, and Dr. Steele. Mr. Munro, C.B., Capt. Shaw, C.B., and Sir Jas. Fraser have been invited to join the committee.

